# Rodents Versus Pig Model for Assessing the Performance of Serotype Chimeric Ad5/3 Oncolytic Adenoviruses

**DOI:** 10.3390/cancers11020198

**Published:** 2019-02-08

**Authors:** Lisa Koodie, Matthew G. Robertson, Malavika Chandrashekar, George Ruth, Michele Dunning, Richard W. Bianco, Julia Davydova

**Affiliations:** 1 Division of Basic and Translational Research, Department of Surgery, University of Minnesota, Minneapolis, 55455 MN, USA; kood0006@umn.edu (L.K.); robe1414@umn.edu (M.G.R.); 2 Department of Pharmacology, University of Minnesota, Minneapolis, 55455 MN, USA; chand374@umn.edu; 3 Experimental Surgical Services, Department of Surgery, University of Minnesota, Minneapolis, 55455 MN; USA; gruthvet@gmail.com (G.R.); dunni003@umn.edu (M.D.); Bianc001@umn.edu (R.W.B.)

**Keywords:** oncolytic adenovirus, Ad5/3, Species B, serotype 3, animal model, preclinical model, replication, pig, porcine, syrian hamsters

## Abstract

Oncolytic adenoviruses (Ad) are promising tools for cancer therapeutics. Most Ad-based therapies utilize species C serotypes, with Adenovirus type 5 (Ad5) most commonly employed. Prior clinical trials demonstrated low efficiency of oncolytic Ad5 vectors, mainly due to the absence of Ad5 primary receptor (Coxsackie and Adenovirus Receptor, CAR) on cancer cells. Engineering serotype chimeric vectors (Ad5/3) to utilize Adenovirus type 3 (Ad3) receptors has greatly improved their oncolytic potential. Clinical translation of these infectivity-enhanced vectors has been challenging due to a lack of replication permissive animal models. In this study, we explored pigs as a model to study the performance of fiber-modified Ad5/3 chimeric vectors. As a control, the Ad5 fiber-unmodified virus was used. We analyzed binding, gene transfer, replication, and cytolytic ability of Ad5 and Ad5/3 in various non-human cell lines (murine, hamster, canine, porcine). Among all tested cell lines only porcine cells supported active binding and replication of Ad5/3. Syrian hamster cells supported Ad5 replication but showed no evidence of productive viral replication after infection with Ad5/3 vectors. Transduction and replication ability of Ad5/3 in porcine cells outperformed Ad5, a phenomenon often observed in human cancer cell lines. Replication of Ad5 and Ad5/3 was subsequently evaluated in vivo in immunocompetent pigs. Quantitative PCR analyses 7 days post infection revealed Ad5 and Ad5/3 DNA and replication-dependent luciferase activity in the swine lungs and spleen indicating active replication in these tissues. These studies demonstrated the flaws in using Syrian hamsters for testing serotype chimeric Ad5/3 vectors. This is the first report to validate the pig as a valuable model for preclinical testing of oncolytic adenoviruses utilizing Adenovirus type 3 receptors. We hope that these data will help to foster the clinical translation of oncolytic adenoviruses including those with Ad3 retargeted tropism.

## 1. Introduction

Oncolytic adenoviruses (Ad) have emerged as a promising new therapy in the fight against cancer. Adenoviruses are a family of non-enveloped DNA viruses comprised of 51 different serotypes organized into 6 species (A–F) [1]. The viral capsid is an icosahedron which is composed of hexon and penton base proteins. Attached to each penton base is a fiber protein which consists of three regions, the tail, the shaft, and the knob regions. Initial binding of these viruses to cells is mediated through interactions between the viral fiber protein and various cell receptors which differ somewhat between the species. After initial binding, all adenoviral species undergo internalization via engaging αν integrins as secondary receptors [2].

Cellular receptors which interact with viral fiber proteins have been extensively studied. The cellular protein, coxsackie and adenovirus receptor (CAR), has been found to be the main receptor implicated in binding of most adenoviral serotypes. Species A and C–F all utilize CAR for initial binding [3,4,5]. Species B adenoviruses comprise two subspecies: the B1 group (Ad3, Ad7, Ad16, Ad21, and Ad50) and B2 (Ad11, Ad14, Ad34, and Ad35). Species B viruses are associated with infection of a variety of human organ systems including the respiratory tract, the urinary tract, the kidney, and the eye [5]. Discovery of the receptors utilized by group B adenoviruses proved to be quite challenging. The membrane regulator protein of complement activation named CD46 was found to bind most of the serotypes of Species B adenovirus [6]. Marttila et al. concluded that CD46 was used by all group B serotypes except Ad3 and Ad7, while Sirena et al. demonstrated that Ad3 also binds CD46 [7,8]. A second receptor, initially known as receptor X and later identified as a cell adhesion molecule called Desmoglein 2 (DSG2), is also implicated in initial binding for Ad3, Ad7, Ad11, and Ad14 [9,10]. CD80 and CD86 have also been reported to play a role in binding, although these are currently thought to be of less importance [11].

The effectiveness of oncolytic adenoviral therapy is greatly dependent on the binding domains of these viral vectors. The majority of conventional Ad-based vectors utilize the human Adenovirus type 5 (Ad5) serotype which belongs to the species C adenoviruses. The Ad5 genome has been well characterized and is easily manipulated. Because species C viruses utilize CAR for initial binding, replication-competent Ad5-based cancer therapeutics are effective for cancers with upregulated CAR expression. Unfortunately, these viral constructs have understandably exhibited low-efficacy in CAR-deficient cancers such as pancreas, gastric, melanoma, prostate, colon, ovarian, breast, and others [12]. To overcome this problem, intensive work has been done to develop Ad-based vectors that can undergo CAR-independent entry.

One strategy which has remarkably improved the oncolytic potential of Ad-based therapies involves formation of viral chimeras designed to accommodate structural components from different virus serotypes. The effectiveness of this approach was first verified in 1996 when the Ad5 fiber knob was replaced with the knob from Ad3 and subsequently demonstrated significant enhancement in virus binding and entry [13]. Since then, infectivity-enhanced vectors utilizing species B binding domains (Ad5/3, Ad5/35, Ad11) have demonstrated remarkable superiority in many cancers relative to the parental Ad5 strain and have revolutionized the field of oncolytic virus therapy [10,14,15,16,17,18,19,20,21,22].

Although the efficacy of these tropism-modified chimeric adenoviral vectors has been clearly established, their translation to clinical practice has been challenging due to the lack of a suitable animal model. Many different models including mice, cotton rats, rabbits, Syrian hamsters, dogs, and pigs have been investigated for even partial support of adenoviral replication. All of these studies have been conducted with species C serotypes (Ad5) with Ad5 type binding domain [23,24,25,26,27]. We were unable to find any literature directly testing replication ability of either Ad5/3 vectors or any species B adenoviral vectors in non-human tissues which was surprising because the utilization of different cellular receptors to increase oncolytic efficacy is well known.

While human adenovirus can effectively transduce a wide range of non-human cell lines, it tends to produce productive viral progeny in human cells only. The standard murine model is generally considered a non-permissive host to Ad5 [27,28]. Other small animals including rabbits, woodchucks, and guinea pigs also showed poor ability to support effective Ad5 replication [23]. A single study reported replication of Ad5 vectors in canine cells although these results have thus far not been reproducible [24].

The extensive search for appropriate animal models which support human adenoviral replication has led to the identification of two important species, cotton rats and Syrian hamsters. Cotton rats were shown in multiple studies to be semi-permissive to human adenoviral infection and oncolytic effect was demonstrated in this animal model [25,29,30]. Their widespread use has been hampered because they are a difficult species to work with and have limited commercial availability [26]. Syrian hamsters have emerged as an essential animal model for evaluation of species C serotypes [26,31,32,33,34,35,36]. These animals have been studied extensively and are known to support Ad5 replication. This immune-competent model has enabled the evaluation of Ad behavior in situ with natural host immune responses.

Hamsters have been used for conducting pre-clinical toxicity studies for many Ad5-based adenoviral vectors. In this capacity, this species has been a vital resource in bringing adenoviral treatments to clinical trial. A few attempts were made to use the Syrian hamsters as a preclinical model for Ad5/3 retargeted vectors [37,38]. Despite the prior use of this model to evaluate these tropism-modified viruses, the ability of the hamster to support replication of species B retargeted vectors has not been directly tested.

Pigs are widely used in other fields of biomedical research because of their similarities to human anatomy, physiology, and genetics; however, they have not been seriously investigated for testing of oncolytic adenoviral vectors. Over the last two decades, only a few groups have studied porcine cell lines for Ad5 replication [23,39,40,41,42,43,44,45]. Jogler et al. took the experiments further, evaluating Ad5 in vivo, and showed evidence of effective replication in pigs [23]. While this study did provide some promising data, it was limited by the early time points used to demonstrate productive viral replication and by the use of only a single animal in each group for the in vivo studies. Difficulty and expense of working with such a large animal with few commercially available cell lines have limited the wide acceptance of this model. As the field has matured and many new chimeric vectors have been created, it may now be beneficial to further pursue this model to help make the jump to clinical translation.

In this study, we set out to test various non-human models including mice, Syrian hamsters, dogs, and pigs for replication potential with both Ad5 and Ad5/3 vectors. We have demonstrated that among all tested non-human cell lines, only porcine cells support both binding and replication of the Ad3-retargeted adenoviral vectors. The in vivo studies in immunocompetent Yorkshire pigs confirmed that the pig is a valid preclinical model for evaluating performance of both Ad5 and Ad5/3 replication competent vectors. We hope that these data will help to foster clinical translation of oncolytic adenoviruses including those with species B retargeted tropism.

## 2. Results

### 2.1. Analysis of Virus-Cell Binding

**Figure 1:**
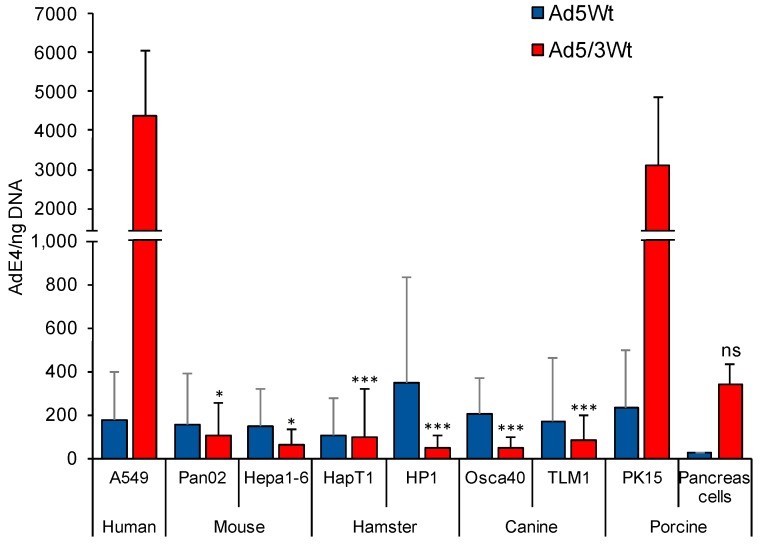
Binding ability of Ad5 and Ad5/3 to non-human cell lines. Human lung adenocarcinoma A549 cells served as Ad5 and Ad3 receptor positive control. Mouse, hamster, canine and porcine cell lines were infected with Ad5 and Ad5/3 for 1h at 4 °C to prevent virus internalization. Isolated total DNA was analyzed by PCR to determine bound Ad (AdE4 gene copy/ng DNA). Amongst the non-human cells, the porcine cells supported the highest levels of Ad5/3 binding. Mouse, hamster, and canine cells demonstrated superior binding with Ad5 vectors versus Ad5/3, while porcine cells and human cell controls showed the opposite trend of Ad5/3 superiority. (* *p* < 0.05; ** *p* < 0.005 *** *p* < 0.0005 Denotes significance to A549 cells).

We first set out to evaluate viral binding of both Ad5 and Ad5/3 vectors in non-human cell lines. Viral binding was quantified using a virus-cell binding assay where cells were infected with virus and then incubated at 4 °C to prevent viral internalization. Total cell DNA was analyzed with PCR to determine the amount of bound viral DNA expressed as AdE4 DNA copies per ng DNA. Binding events in mouse, hamster, canine, and porcine cell lines were compared to human lung adenocarcinoma A549 which is used as an Ad5 and Ad3 receptor positive control (Figure 1).

Whereas infection with Ad5 resulted in similar levels of binding activity in all tested non-human and human cell lines, infection with Ad5/3 varied remarkably between the species. Namely, binding with Ad5/3 virus in murine Pan02 and Hepa1-6 was approximately 40-fold and 65-fold lower respectively when compared to human A549 (*p* < 0.00001, *p* < 0.000003 respectively). Viral DNA copy numbers in hamster cells were 40 to 80-fold lower than in A549 (*p* = 0.00004). Canine Osca40 and TLM1 cells showed a similar tendency, demonstrating a significantly lower binding ability of Ad5/3 (*p* < 0.00001). Porcine PK15 cell lines were the only cells to demonstrate viral binding events at a rate near the A549 human control (3095 ± 1750.24). The primary swine cells bound Ad5 vectors quite poorly but had the second highest number of Ad5/3 binding events among non-human cell lines behind PK15 cells. Notably, pig cells were the only non-human cells tested to show stronger binding of Ad5/3 vectors than Ad5 vectors, a pattern seen in A549.

### 2.2. Analysis of Gene Transduction

**Figure 2:**
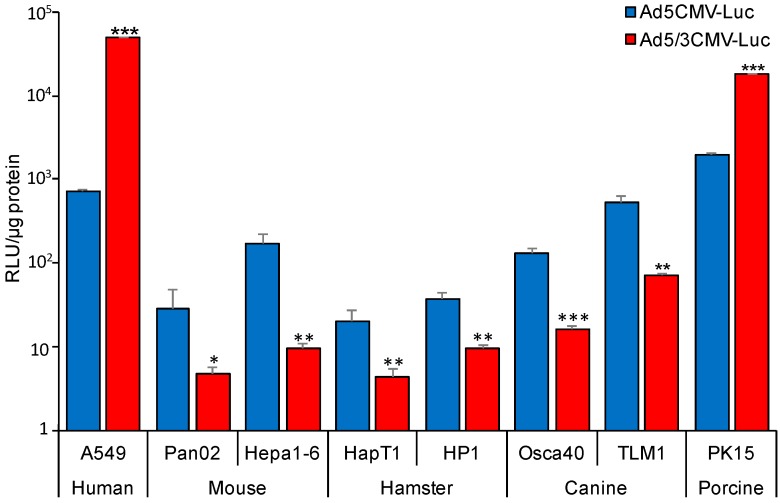
Gene transduction of Ad5 and Ad5/3 in non-human cells. Mouse, hamster, canine, and porcine cells along with human controls were infected with replication-deficient Luciferase expressing vectors, Ad5CMV-Luc and Ad5/3CMV-Luc. Luciferase activity was determined 2 days post infection. Infection with Ad5 efficiently transduced all non-human cells. Compared to Ad5, Ad5/3 gene transfer was significantly lower in rodent and canine cells but higher in porcine cells (* *p* < 0.05, ** *p* < 0.005, *** *p* < 0.001 Denotes significance to Ad5 infected cells).

We next tested the cell lines to evaluate adenoviral transduction efficiency. A pair of identical replication deficient Luc-expressing vectors with either wild type fiber (Ad5CMV-Luc) or chimeric Ad5/3 fiber (Ad5/3CMV-Luc) was used to infect the cell lines, then Luc activity was used as a measure of gene transfer (Figure 2). Rodent and canine cells show a significant difference in gene transduction between Ad5 and Ad5/3 vectors, with Ad5 viruses being consistently more effective at gene transfer. Murine Pan02 and Hepa1-6 demonstrated significantly lower levels of transgene expression with the Ad5/3 chimera being 6.1-fold and 17.8-fold lower than that of Ad5 (*p* = 0.048 and 0.003), respectively. Similarly, hamster HapT1 and HP1 cell lines showed very poor gene transfer with Ad5/3 Luc activity being 4.69-fold and 3.92-fold lower respectively relative to Ad5. Of note, compared to human control, all four rodent cell lines had negligible luciferase activity with Ad5/3 vectors, nearly 4 orders of magnitude lower than in A549. The trend of significant superiority of Ad5 versus Ad5/3 was also observed in canine cells. Conversely, porcine PK15 cell lines demonstrated significantly increased transduction efficiency with Ad5/3 vectors compared to Ad5 viruses (*p* < 0.0004). Additionally, RLU levels of porcine cells approached those seen in human cells.

### 2.3. Replication-Mediated Cell Killing

**Figure 3:**
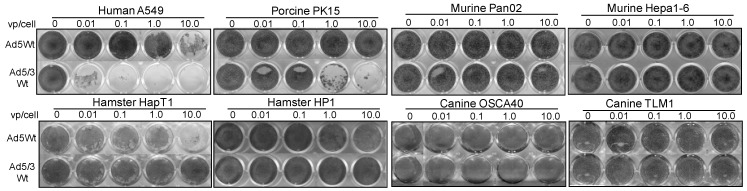
Replication-dependent cytocidal ability of Ad5 and Ad5/3 in non-human cells. The ability of Ad5 and Ad5/3 to replicate and cause a cytocidal effect in non-human cells was analyzed by crystal violet assay. Human A549 adenocarcinoma cell line was used as a replication-permissive control. The cells were infected with low titers (0.01, 0.1, 1, and 10 viral particle/cell) to allow at least a few cycles of virus replication. No cytocidal activity was observed in mouse and canine cells. Low titers of Ad5 infection effectively killed hamster cells HP1 (1–10 vp/cell) and HapT1 (10 vp/cell). Cell death after infection with Ad5/3 was observed only in porcine PK15 cells (1–10 vp/cell) and human A549 controls (0.01–10 vp/cell).

To determine whether tested non-human cell lines can support viral replication, we infected cells with replication-competent Ad5 and Ad5/3 vectors (Ad5Wt and Ad5/3Wt). The cells were infected with low titers (0.01, 0.1, 1, and 10 viral particle (vp)/cell) for 8 days to allow at least a few cycles of virus replication. The surviving cells were stained with crystal violet (Figure 3). We observed no cell killing in murine cells with either vector. Hamster cells presented evidence of replication-mediated cell death when infected with Ad5 vector; however, we did not observe any cytolysis after Ad5/3 infection. Canine cells were resistant to viral cytolysis with both viral constructs and stayed intact for the entire duration. PK15 porcine cell line demonstrated remarkable adenovirus-mediated cell killing upon infection with low titers of Ad5/3 virus, however no significant oncolysis was observed with low titers of its Ad5 counterpart.

**Figure 4:**
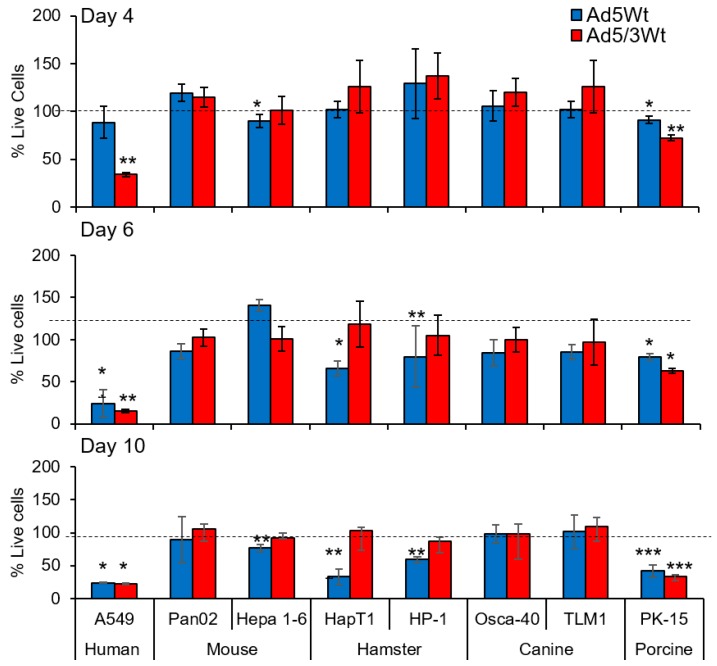
Quantitative cell viability analysis after infection with Ad5 and Ad5/3. All cell lines were infected with either Ad5 or Ad5/3 wild type replication viruses. Cell viability (% live cells) was assessed with MTS assay 4, 6, and 10 days post infection. Murine Pan02, hamster, and porcine cell lines showed significant reduction in viability after Ad5 infection. Infection with Ad5/3 retargeted viruses caused significant cell death only in porcine PK15 cells and A549 human controls (* *p* < 0.05; ** *p* < 0.0001; *** *p* < 0.000001 Denotes significance to uninfected cells).

To quantify the cytolytic effect, all cell lines were plated and infected with Ad5 and Ad5/3 vectors at a titer of 100 vp/cell and incubated for 4, 6, and 10 days. MTS cell viability assay was then performed to quantify cell death (Figure 4). Murine cells were killed when infected with Ad5, particularly the Hepa1-6 line which showed a significant amount of cell death. We did not observe any significant cytolysis in murine cells after Ad5/3 infection. Canine cells showed no significant cell death with either viral vector. Hamster cells were killed quite efficiently with Ad5 vectors but again, no killing effect is seen when infected with serotype chimeric Ad5/3 vectors. Porcine PK15 cells were killed by both Ad5 and Ad5/3 vectors with the latter being more efficient than its Ad5 counterpart at earlier time points.

### 2.4. Analysis of Replication-Dependent Gene Expression

**Figure 5:**
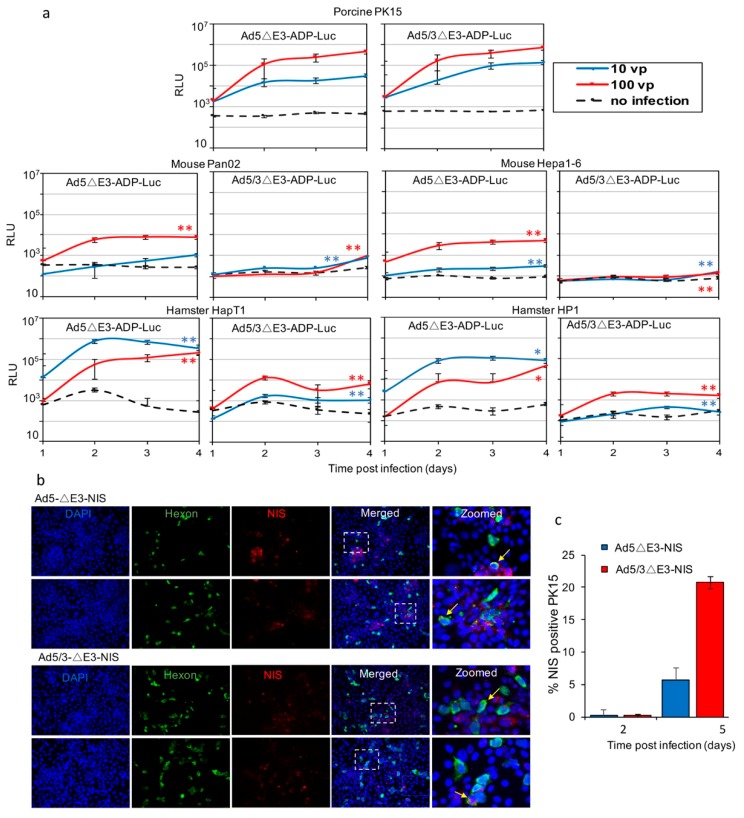
Gene expression as an indicator of virus replication in non-human cell lines. (**a**) Rodent and porcine cells were infected with Ad5 and Ad5/3 viruses which express luciferase in a replication-dependent manner (Ad5ΔE3-Luc and Ad5/3ΔE3-Luc). Luc activity was determined on days 1, 2, 3 and 4 post infection. Hamster and porcine cells demonstrated time-dependent increase in Luc expression after Ad5 infection, denoting active replication. Only porcine cells showed time-dependent increase in Luc activity after Ad5/3 infection. Trend analysis across the cell lines shows significant difference in rate of change relative to porcine PK15 under same conditions (* *p* < 0.05; **p < 0.005). (**b**) Immunofluorescent images of porcine PK15 cells infected with Ad5 and Ad5/3 vectors expressing replication-dependent sodium-iodide-symporter (NIS) and stained for Ad-hexon (green) and NIS (red) proteins, co-localization indicated in yellow (see arrows), mag. 20×. (**c**) Quantitative assessment by Flow Cytometry of PK15 porcine cells expressing NIS. From day 2 to day 5 the percentage of NIS-positive cells increased after infection with both Ad5ΔE3-NIS and Ad5/3ΔE3-NIS. The percentage of NIS-positive cells after infection with Ad5/3 was 4-fold greater than with Ad5 (*p* < 0.05).

In order to further demonstrate and better quantify replication ability of adenoviral vectors in tested cell lines, we next evaluated replication-dependent gene expression. Porcine, mouse, and hamster cells were infected with replication-competent Ad5- and Ad5/3-ΔE3-Luc vectors with the Luc gene placed in the Ad E3 region (Figure 5a). We have previously reported that in this vector structure, expression of the reporter transgene follows a late profile due to control by the major late promoter and is therefore consistent with the replication cycle [46].

Murine cells had low luciferase activity after infection with either Ad5 or Ad5/3 vectors. We did not observe any time dependent increase in Luc activity over time indicating a lack of viral replication. Hamster cell lines did demonstrate effective replication-dependent gene expression with Ad5 vectors (HP1 10 vp; Day 1–174 RLU, Day 4–49,432 RLU) (HapT1 10 vp; Day 1–909 RLU, Day 4–213,239 RLU). However, Luc activity reached relatively low levels when these same cells were infected with Ad5/3 viruses (HP1 100 vp; Day 4–1678 RLU) (HapT1 100 vp; Day 4–6331 RLU). Porcine PK15 cells demonstrated replication-dependent gene expression after infection with both Ad5 and Ad5/3 viruses. Infection of porcine PK15 cells with Ad5/3 vectors showed a particularly robust increase of luciferase activity over time (10 vp; Day 1–2822 RLU, Day 4–144,896 RLU).

We performed trend analysis to evaluate the rate of change of all tested cell lines compared to PK15. Murine cells demonstrated a significantly lower rate of change (*p* < 0.02) except for Pan02 cells infected with Ad5 at 10 vp/cell which trended lower (*p* = 0.28). Hamster cells infected with Ad5/3 showed a significantly lower rate of change compared to PK15 under the same conditions (*p* < 0.001). HapT1 and HP1 cells infected with Ad5 at 10 vp/cell had a significantly higher rate of change (*p* < 0.01) which is not unexpected as it is known that hamster cells support productive viral replication with Ad5 vectors.

To further assess porcine cells, we tested another pair of Ad vectors which express the human sodium iodide symporter (NIS) in replication-dependent manner from the E3 region (Ad5- or Ad5/3-ΔE3-NIS). After infection, cells were stained with fluorescent-conjugated antibodies for NIS and Ad hexon proteins. In fluorescent captured images (Figure 5b), we observed expression of both Ad hexon and NIS in PK15 cells infected with both Ad5 and Ad5/3 viruses. Quantitative assessment of NIS positive PK15 cells using flow cytometry analysis (Figure 5c) revealed similar levels of NIS-positive cells 2 days after infection with either Ad5- or Ad5/3. By 5-days post infection, after giving the virus sufficient time to replicate, we observed that the percentage of NIS-positive PK15 cells after Ad5/3 infection was 4-fold greater than that observed after Ad5-fiber unmodified infection (*p* < 0.05). These in vitro experiments are the first to demonstrate the ability of porcine cells to support replication-dependent gene expression associated with Ad5/3-retargeted vectors and even suggest its superiority over Ad5 vectors.

### 2.5. In Vivo Analysis of Ad5 and Ad5/3 Replication in Pigs

**Figure 6:**
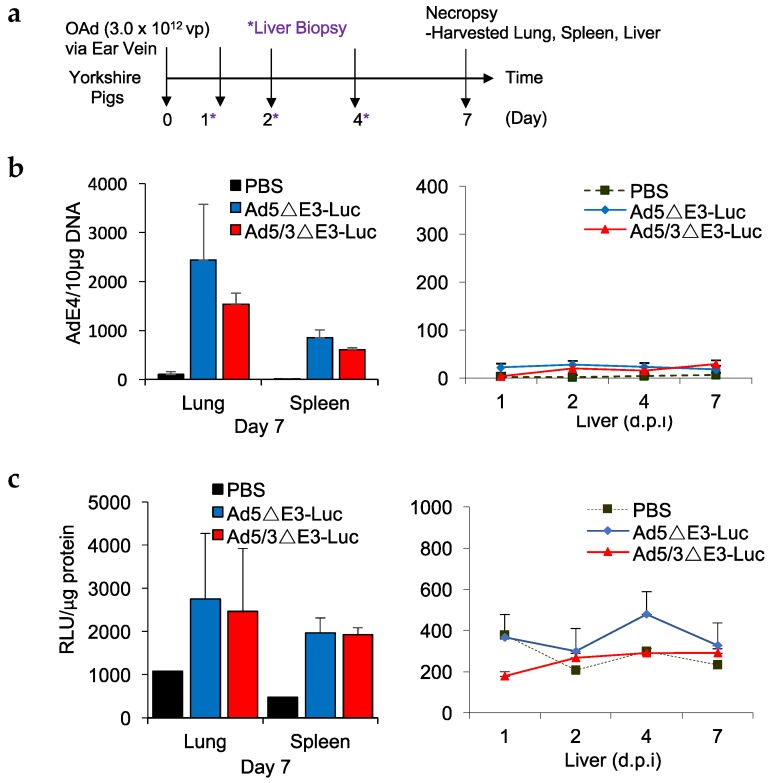
In vivo evaluation of Ad5 and Ad5/3 replication in pigs. (**a**) Pigs were injected with a single dose of Ad5∆E3-Luc or Ad5/3∆E3-Luc at 3 × 10^12^ vp via ear vein. Liver biopsies were performed at days 1, 2, and 4 post infection, primary organs were harvested 7-days post infection. (**b**) Viral DNA was present in lungs and spleen, but negligible copy numbers were measured in liver samples. Viral DNA copy numbers correlated well with replication dependent Luc expression. (**c**) Luc activity was detected in lungs and spleen. No significant replication-dependent Luc expression was observed in liver tissues.

To assess swine as a preclinical model to study replication-competent human adenoviral vectors, we systemically injected immunocompetent Yorkshire pigs with 3 × 10^12^ vp of fiber-unmodified (Ad5-ΔE3-Luc) or Ad5/3-modified (Ad5/3-ΔE3-Luc) Luc expressing replicative virus. Liver biopsies were obtained on days 1, 2, and 4. Necropsy was performed on day 7 and major organs were evaluated for viral infection. Seven days post infection with either vector, adenoviral DNA was detected in the pigs’ lung and spleen (>1000 AdE4 copy/µg DNA) (Figure 6a). The viral DNA quantity in lungs and spleens correlated well with replication-dependent Luc expression (~2500 RLU/µg and ~2000 RLU/µg, respectively), measuring similar levels of Luc activity with both Ad5- and Ad5/3-∆E3-Luc (Figure 6b). Interestingly, unlike in mice, no liver tropism was observed in pigs. Viral replication in liver tissues was almost negligible, demonstrating much lower levels of viral DNA in liver biopsies at earlier time points (<30.0 AdE4 copy/µg) and background values 7 days post infection. Again, DNA copy numbers correlated well with the Luc expression in liver biopsies. These results confirm the ability of adenoviral vectors to actively replicate in live swine after systemic administration, further supporting the usefulness of pigs as a potential model testing adenoviral vectors, including species B-retargeted viral constructs.

### 2.6. Analysis of Systemic Toxicity in Pigs after Systemic Administration of Adenoviral Vectors

No significant signs of toxicity were observed in either Ad5 or Ad5/3 infected pigs after administration of a single dose of 3 × 10^12^ viral particles. Aspartate aminotransferase (AST) and lactate dehydrogenase (LDH), which may increase in both liver and lung damage, showed some elevation in the Ad5/3 group on day 4 (both of them within the reference interval) and returned to baseline at day 7. The liver damage marker sorbitol dehydrogenase (SDH) showed mild elevation at day 7 in the Ad5 group, however, it was not clinically relevant. Alkaline phosphatase (ALP) and liver specific alanine aminotransferase (ALT) was normal in all pigs (See Table 1 for the complete serum chemistry profile of individual pigs). No microscopic changes in the kidney or liver were seen upon necropsy 7 days post infection. Microscopic changes were limited to lungs and included interstitial pneumonia; however, this pneumonia was observed in both experimental and control groups. Analysis of the hematology parameters in control and infected pigs at baseline and post infection revealed an increase within the reference range in white blood cells (neutrophils and monocytes) on day 2 that returned to baseline by day 7 (Table 2). No significant changes were observed in red blood cell or platelet levels.

## 3. Discussion

As oncolytic adenoviral technology has matured, engineering of tropism-modified viruses utilizing species B (Ad3, Ad35, Ad11) binding domains has emerged as a crucial step to improve the efficacy of conventional Ad5-based vectors. This retargeting has improved infectivity of oncolytic adenoviral therapeutics across a broad range of tumors including pancreatic, gastric, ovarian, melanoma, esophageal, breast, prostate, and others [10,14,15,16,17,18,19,20]. As the field now moves to bring tropism-modified viruses to clinical practice, the appropriateness of current preclinical animal models must be questioned, and a species B receptor permissive species identified.

Preclinical toxicology studies require adenovirus replication-permissive animal models in which the effect of Ad-induced cytolysis and immune response can be evaluated. In order to pinpoint a potential preclinical model for species B-retargeted Ads, we have performed a comparative analysis of the replication properties of chimeric Ad5/3 vector in various non-human cell lines (murine, Syrian hamster, canine, and porcine). We found little evidence of productive viral infection with either Ad5 or Ad5/3 vectors in both murine and canine cell lines. Additionally, the tropism modified viruses uniformly underperformed their wild type counterparts in both of these species.

Immunocompetent Syrian hamsters are semi-permissive for human Ad5 replication and have been proven to work well for preclinical evaluation of Ad5-based viral constructs, including RGD-modified vectors [26,31,32,33]. Interestingly, a few preclinical studies have begun using the hamster model to assess Ad5/3 vectors in Syrian hamsters; even though permissiveness of hamster models for these and other tropism-modified vectors was never formally established [37,38]. Because of the difficulty in establishing an animal model for Ad5 vectors, coupled with the known difference of Ad3 receptors, we felt that it was essential to directly address the Syrian hamster’s ability to undergo productive infection with chimeric serotype Ad5/3 vectors. Our studies suggest that Syrian hamsters are far less permissive for Ad5/3 than for Ad5. While we confirmed clear evidence of Ad5 replication and replication-dependent cell death in hamster cells, similar results were absent in cells infected with Ad5/3 vectors. Poor infectivity after Ad5/3 infection of Syrian hamster cells has been reported in literature by others [47].

We suggest that this discrepancy could be explained by the lack of CD46 and DSG2 receptors on the rodent cells. Indeed, a few groups have reported CD46 expression in mice and rats is limited to the testes [48,49]. Our binding and gene transfer assays, which directly assess viral entry, demonstrated very low viral entry and negligible transduction efficiency in hamster cells infected with Ad5/3 viruses. When directly compared to human A549 control cell line, hamster cells infected with Ad5/3 showed 40 to 80-fold lower virus biding and at least 5000-fold lower transduction efficiency. A complete and more detailed analysis of Ad5 (CAR) and Ad3 (CD46 and DSG2) receptor expression in non-human cell lines is needed to further elucidate the reasons that lead to differences in infectivity. However, due to major differences in the nucleotide sequences between species, a single primer or antibody will be insufficient to objectively compare the receptor expression pattern. Therefore, we believe that the functional binding assay provides the most accurate evaluation of the binding ability of Ad5/3 vectors in non-human species. Cell viability and replication assays showed no Syrian hamster cell death with Ad5/3 viruses. When compared to constructs expressing Ad5 binding domains, replication dependent gene expression was nearly two orders of magnitude lower when hamster cells were infected with viruses expressing Ad3 binding domains. This series of experiments convincingly demonstrates that Syrian hamsters are not a permissive host for productive infection with Ad5/3 adenoviral vectors and as such, these animals cannot provide meaningful preclinical data.

Of our tested cell lines, porcine cells were the only non-human cells to demonstrate susceptibility to infection with tropism modified adenoviral vectors. In each of the experiments, we saw improved performance of Ad5/3 viruses compared with Ad5 viruses in porcine cells. This pattern mimicked that seen in human controls. Each of the other cell lines (rodent and canine), exhibited an opposite pattern, with Ad5 constructs outperforming Ad5/3 in each of the five in vitro experiments. Additionally, Ad5/3 infected porcine cells were significantly more efficient at expression of replication dependent NIS expression, with three times more cells testing positive for NIS expression in the Ad5/3 vs. Ad5 cells.

We note that due to the lack of available porcine cancer cell lines on the current market, the porcine cells used here are normal (non-carcinogenic), while other cell lines tested represent malignancy. Human adenoviruses possess an intrinsic selectivity for replication in cancer tissues and tend to show an increase in virus infectivity towards malignant cells vs. normal cells [50]. The level of both Ad5 and Ad5/3 adenovirus replication observed in normal porcine cells in comparison with human A549 control suggest that malignant porcine cells would support the replication of human Ad5/3 with similar efficiency to malignant human cells.

While in vitro studies are an important first step, in vivo testing in immunocompetent animals is needed to truly evaluate validity of the pig model. We administered Ad5 and Ad5/3 viruses expressing replication-dependent luciferase to immunocompetent Yorkshire pigs and evaluated virus distribution 7 days post infection. PCR analyses of viral DNA revealed significant presence of both Ad5 and Ad5/3 in the lungs and spleens of these animals. DNA copy numbers were similar between animals infected with Ad5 vectors and those infected with Ad5/3. Because this viral DNA could potentially be secondary to sequestration of the inoculated viral particles and not actually from viral replication, we have examined the tissues for replication-dependent luciferase expression. The pig tissues exhibited the same pattern of Luc expression, with the highest levels of transgene being seen in the lungs and spleen. Again, the levels of gene expression between the two different viral constructs were not significantly different. These data provide strong evidence of active viral replication with production of functional progeny versus simple sequestration of viral inoculum. This knowledge supports the feasibility of using pigs as a model to study species B retargeted adenoviruses.

Adenoviruses have a well-known tropism for the liver in rodent models and we were interested in discovering a similar phenomenon in pigs. To study this, in addition to the day 7 analysis, liver biopsies were taken from the injected pigs at multiple time points to analyze both viral DNA and replication-driven luciferase expression. Interestingly, the levels of viral DNA and Luc activity in liver were almost negligible throughout the experiment, indicating a very different pattern of adenoviral biodistribution in pigs compared to rodents. No liver toxicity was observed in pigs despite a very high dose (3 × 10^12^ vp) of systemically administrated virus; and in general, the adenovirus-injected pigs remained in good health and condition demonstrating no adenovirus-related toxicity. Additional studies with multiple time points are necessary to further elucidate the biodistribution and pharmacokinetics of systemically administered adenoviruses in the pig model.

These studies have demonstrated the flaws of using Syrian hamsters for testing serotype chimeric Ad5/3 vectors. This also is the first report to validate the pig as a valuable model for preclinical testing of oncolytic adenoviruses utilizing Adenovirus type 3 receptors. Immunocompetent pigs, which more closely mimic human anatomy and physiology, will be able to provide useful information on the biodistribution and toxicity of these adenoviral vectors and may help to bridge the gap to clinical translation.

## 4. Materials and Methods

### 4.1. Cell Lines and Cell Culture

Cell lines were obtained from American Type Culture Collection (Manassas, VA, USA) unless otherwise specified and included human lung adenocarcinoma (A549); murine pancreatic adenocarcinoma, Pan02 and hepatoma, Hepa1-6; Syrian hamster pancreatic cancers, HP1 and HapT1; (a kind gift of Dr. M.A. Hollingsworth, University of Nebraska, Lincoln, NE, USA), canine osteosarcoma, Osca40 [51] and canine melanoma, TLM1 [52] (both a kind gift of Dr. J. Modiano, University of Minnesota, Minneapolis, MN, USA) and non-malignant porcine kidney epithelial, PK15. All human and non-human cells were cultured at 37 °C with 5% CO_2_ under humidified conditions. Growth medium was supplemented with 5–10% fetal bovine serum, 1% penicillin-streptomycin and were maintained as adherent monolayers. A549, Pan02, Hepa1-6, HP1, and were cultured in DMEM. PK15 was cultured in EMEM. Osca40 and TLM1 cells were grown in DMEM supplemented with 5 mM Hepes (Sigma, St. Louis, MO, USA). Primary swine pancreas cells were isolated in our lab and cultured in EMEM supplemented 20% FBS + 20 ng/mL EGF + 25 µg/mL Bovine pituitary extract + 1% Pen strep/ampicillin. Briefly, swine pancreas was surgically removed, and pancreatic ducts were dissected free of tissues. Duct was then digested with 10 mg/mL collagenase-IV and 0.25% trypsin shaking for 4 h at room temperature. Digested ducts were filtered using a 70 µM cell strainer and whole cells were plated onto collagen I coated dishes. Pancreatic duct cells outgrew by 9–14 days, colonies were consequently maintained and sub-cultured for use in experiments. Duct cells were confirmed to express EpiCAM and Cyk19.

### 4.2. Adenoviral Vector Construction

Three pairs of adenovirus type 5 (Ad5) and the chimeric vectors with the Ad5/3-modified fiber (Ad5/3) were used in this study. All Ad5/3 vectors had a chimeric fiber composed of the Ad5 shaft and the Ad3 knob which was incorporated as we described previously [14]. In the binding and killing assays we used replication-competent wild type Ad5 (Ad5Wt) and its retargeted counterpart with the Ad5/3 modified fiber (Ad5/3Wt) [14]. To assess the gene transfer, we used a pair of replication-deficient vectors with the luciferase (Luc) inserted under control of the CMV promoter (Ad5CMV-Luc and Ad5/3CMV-Luc) [46]. To assess replication-dependent gene transfer, we used replication competent vectors expressing Luc or Sodium Iodide Symporter (NIS) (Ad5Wt-∆E3-Luc, Ad5Wt-∆E3-NIS, respectively). These viral vectors were constructed based on the ∆E3 structure as was described previously [46,53]. Briefly, we have deleted the non-essential genes within the AdE3-region (∆E3) while maintaining the Adenoviral Death Protein (ADP) gene. Replication-competent viruses were propagated in A549, while 911 cells were used to amplify replication-deficient counterparts. The viral vectors were purified by double CsCl density gradient ultracentrifugation, followed by dialysis against phosphate-buffered saline (PBS) with 10% glycerol. Virus preps were stored in aliquots at −80 °C until used.

### 4.3. In Vitro Analysis of Ad5 and Ad5/3 Binding Ability

Cells (5.0 × 10^4^ cells/well) grown in 24 well plates and infected with Ad5Wt or Ad5/3Wt at 2000 viral particle (vp) per cell for 1.5 h at 4 °C to prevent virus internalization. Unbound virus was washed away, and cells were collected by scraping. Total DNA was extracted using the Qiagen DNA Blood and Tissue Kit (Qiagen, Germantown, MD, USA, Cat. No. 69504) and DNA concentration determined using a Nanodrop spectrophotometer (Thermofisher, Minneapolis, MN, USA). PCR using primers for the AdE4 gene was used to determine the bound virus in total DNA. The relative AdE4 gene copy number in samples were calculated using a standard curve with known concentrations of the E4 gene as previously described [54].

### 4.4. In Vitro Analysis of Ad5 and Ad5/3 Gene Transduction

Cells (5.0 × 10^4^ cells/well) grown in 24 well plates and infected with replication-deficient Ads expressing luciferase (Ad5CMV-Luc and Ad5/3CMV-Luc) at 500 vp/cell for 2 h. Infection medium was removed, and infected cells cultured in appropriate growth medium. Luciferase activity (represented as relative light units, RLU) was determined 2-days post infection using the Luciferase Assay Kit per manufacturer’s instructions (Cat# E1300, Promega, Madison, WI, USA). Protein estimation was performed using the DC Assay (BioRad, Hercules, CA, USA, cat no. 5000112). The data is expressed as RLU/per µg of total protein.

### 4.5. In Vitro Analysis of Ad5 and Ad5/3 Replication Ability or Cytocidal Effect

Cells (2 × 10^5^ cells/well) grown in 12 well plates were infected with replication competent Ad5Wt and Ad5/3Wt for 2 hours. Infection medium was removed, and infected cells cultured in appropriate growth medium. Human, mouse, hamster and porcine cells were infected at (0, 0.01, 0.1, 10, 100 vp per cell) for 7–12 days. Cells were fixed in 10% buffered formalin for 10 minutes and stained with a 1% crystal violet in 70% ethanol for 20 minutes. Plates were gently rinsed in tap water and air dried before being scanned and converted to grayscale in Adobe Photoshop.

### 4.6. Cell Viability Assay

Cells (3000 cells/well) grown in 96 well plates were infected with replication-competent Ad5Wt and Ad5/3Wt at 10 and 100 vp per cell in 2.5% FBS containing infection medium. After 4, 6, 8 and 10-days post infection, the total cell viability was determined using Cell Titer 96 One Solution Cell Proliferation Assay Kit (Promega, Cat. No G3580). The data is expressed as % live cells relative to untreated control cells.

### 4.7. In Vitro Analysis of Ad Replication Dependent Gene Expression in Porcine Cells

For analysis of Luciferase gene expression, we used a replication-competent Ad5-∆E3-Luc, and its identical counterpart Ad5/3-∆E3-Luc. Mouse, hamster and porcine cells were cultured in 24 well-plates (5.0 × 10^4^ cells/well) and infected with at 10 and 100 vp/cell for 4 h. Infection medium was removed, and infected cells cultured in appropriate growth medium. Luciferase activity was assayed in untreated and virus treated cells 1, 2, 3, and 4 days post-infection using the Luciferase Assay kit is described above. The data is represented as relative light units (RLU). For qualitative analysis of NIS gene expression, we used a replication competent Ad5∆E3-NIS, and identical counterpart Ad5/3∆E3-NIS [55,56]. Porcine cells were cultured in chamber slides (25,000 cells per well) and infected with Ad5- and Ad5/3∆E3-NIS at 10 vp/cell for 24 h. Unbound virus was washed away, and cells fixed in 4% Paraformaldehyde Solution. Fixed cells permeabilized on ice with 0.25% Triton-X100 (Sigma Chemicals cat. no) were the blocked in 2.0% BSA before primary antibody incubation at 4 °C overnight (NIS: Anti-FP5A (Thermo Fisher Scientific, Minneapolis, MN, USA Cat no. MA5-12308 at 1:500) and Anti-Ad-Hexon FiTC-conjugated Millipore (Massachusetts, USA, Cat no. AB1056F) at 1:1000. Mouse Anti-IgG-PE conjugated secondary antibodies (cat no. A32727) were incubated for 1 h at room temperature to anti-FP5A. Slides were cover-slipped with DAPI Anti-fade solution (Vector Labs, CA, USA) before image capturing using a fluorescence microscope (Leica, Minneapolis, MN, USA). For quantitative analysis of NIS gene expression, we used the same NIS expressing vectors to infect Porcine cells cultured in 6 well plates (2.5 × 10^5^ cells per well) and infected with Ad5- and Ad5/3Wt-∆E3-NIS at 10 vp/cell for 2 h. Unbound virus was washed away, and infection media replaced with fresh growth media. Cells were collected by scraping 2- and 5 days post infection and fixed in 4% Paraformaldehyde Solution before staining for NIS expression. NIS stained cells were analyzed on a BD 8-color Flow cytometer (BD Biosciences, San Jose, CA, USA). The data is expressed as the % of NIS expressing cells in 10,000 events.

### 4.8. In Vivo Analysis of Adenovirus Replication in Pigs

Immunocompetent Yorkshire pigs (20–25 kg) were pre-anesthetized with telazol/xylazine, anesthetized with nembutal, and intubated with a cuffed endotracheal tube in a standard surgical suite. A single viral dose of Ad5∆E3-Luc, or Ad5/3∆E3-Luc at 3 × 10^12^ vp was injected via a peripheral ear vein (1 pig per group). Control pig received PBS containing 7% glycerol. Percutaneous liver biopsies were performed on sedated pigs with a true-cut biopsy needle to analyze Ad distribution at days 1, 2, and 4 post-infection. On day 7, all pigs were euthanized with beuthansia D, and liver, lungs and spleen tissues were immediately harvested and cut in half at the center. Half of tissue samples were fixed with buffered formaldehyde for immunostaining and pathological analysis, the second half was snap-frozen and stored at −80 °C until the total DNA was extracted from ~25 mg of tissues. PCR was used to determine the E4 viral DNA copy number as an indication of virus distribution in 1ug of DNA using a standard curve with known E4 gene copies. Luciferase activity was determined in spleen, lung and liver as an indication of viral replication. A complete blood count test (CBC) and blood chemistry analysis was performed on blood serum at Marshfield laboratory (Madison, WI, USA) [57,58]. The clinical pathology and histopathology analyses were performed by an independent Board-Certified Veterinary Pathologists (Experimental Surgical Services, Surgery Department, University of Minnesota, Minneapolis, MN, USA).

### 4.9. Statistical Analysis

Statistical analyses in vitro and in vivo were performed with the Excel data review function (Microsoft 2016). The paired sample t-test was used for comparing the effects of Ad5 and Ad5/3 in the gene transfer assay in the human and non-human cell lines as well as in the porcine tissues. Results with a two-tailed *p*-value < 0.05 were considered statistically significant. The data are presented as the mean values with error bars representing the standard deviation.

We performed trend analysis on the replication dependent gene expression experiments to evaluate the rate of change of all tested cell lines under different conditions (Ad5, Ad5/3, 10 vp/cell, 100 vp/cell). Four linear mixed models (for each virus/dose combination) were fit to compare the rate of change from day 1 to day 4, among five cell lines in each condition. Each model included fixed effects for cell line, day, and their interaction. A random intercept was included for each sample (*n* = 3 per cell line). The outcome variable was log transformed to stabilize its variance across multiple time measurements. All comparisons were relative to PK15 change in log RLU per day.

### 4.10. Ethical Approval

The current studies were approved by the Institutional Biosafety Committee (IBC), Protocol ID: 1601-33271H (PI: Davydova, Julia) and the Institutional Animal Care and Use Committee (IACUC), Protocol ID: 1612-34382A.

## 5. Conclusions

These studies have demonstrated the flaws of using Syrian hamsters for testing serotype chimeric Ad5/3 vectors. This also is the first report to validate the pig as a valuable model for preclinical testing of oncolytic adenoviruses utilizing Adenovirus type 3 receptors. We hope that these data will help to foster clinical translation of oncolytic adenoviruses including those with Ad3 retargeted tropism.

## Figures and Tables

**Table 1:** Serum chemistry. Data from a comprehensive metabolic panel describing the chemical enzyme changes for individual pigs, days post infection (d.p.i).

Serum Chemistry	Control/PBS (d.p.i)				Ad5ΔE3-Luc (d.p.i)				Ad5/3ΔE3-Luc (d.p.i)				Reference
Measurement	0	2	4	7	0	2	4	7	0	2	4	7	Range
Glucose (mg/dL)	136	104	99	97	108	150	88	94	111	120	92	90	85–150
SDH (U/L)	35.3	4.8	3.4	22.3	12.6	10.4	8.7	82.6	24.4	8.6	18.8	12.6	0–45
Total Bilirubin (mg/dL)	0.1	0.1	0.1	0.1	0.1	0.1	0.2	0.1	0.1	0.1	0.1	0.1	0–1
Cholesterol (mg/dL)	91	71	78	88	72	83	89	80	102	106	110	94	36–54
Total protein (g/dL)	5.3	5.6	5.5	5.6	4.5	5.1	5.3	5.6	5.5	5.1	5.6	5.6	7.9–8.9
Albumin (g/dL)	3.5	3.7	3.9	4	3.3	3.7	3.7	4	3.5	3.3	3.6	3.7	1.9–3.3
Urea N (mg/dL)	10	14	9	9	7	11	7	9	6	12	8	8	10–30
Creatinine (mg/dL)	0.9	0.9	0.7	0.8	0.6	0.6	0.5	0.7	0.8	0.8	0.6	0.6	1.0–2.7
Phosphorous (mg/dL)	9.1	8	7.3	6.9	9	8.6	7.9	9.1	8.9	8.4	8.1	8.5	5.3–9.6
Calcium (mg/dL)	10.2	11.1	10.5	10.7	10.4	10.8	10.4	11.2	10.7	10.4	10.9	10.6	7.1–11.6
Sodium (mmol/L)	142	145	144	142	142	145	143	142	142	145	143	141	135–150
Potassium (mmol/L)	3.8	4	4.3	3.8	3.9	4.9	4	4	3.7	4.5	4.1	4	4.4–6.7
Chloride (mmol/L)	104	109	107	102	105	102	103	97	102	105	103	98	94–106
Bicarbonate (mmol/L)	29	27	31	32	32	33	32	25	31	32	31	36	18–27
CK (U/L)	466	1082	1567	2103	1035	732	1247	1078	513	531	2761	1049	61–1251
Gamma-GT (U/L)	42	38	40	43	40	33	33	39	30	27	35	35	10–60
Globulin (g/dL)	1.8	1.9	1.6	1.6	1.2	1.4	1.6	1.6	2	1.8	2	1.9	5.3–6.4
A/G Ratio	1.9	1.9	2.4	2.5	2.8	2.6	2.3	2.5	1.8	1.8	1.8	1.9	0.37–0.51
Urea/Creat ratio	11.1	15.6	12.9	11.3	11.7	18.3	14	12.9	7.5	15	13.3	13.3	N/A
ALP (VALKP) (U/L)	221	205	195	187	259	190	164	203	250	167	190	197	118–395
ALT (GPT) (U/L)	59	61	70	75	68	61	64	77	72	60	74	81	31–58
AST (GOT) (U/L)	37	47	65	56	61	38	54	56	43	33	83	51	32–84
LDH (VLDH) (U/L)	706	654	640	683	654	615	728	756	693	501	782	536	380–634
Triglycerides (VTRIG)	37	45	37	65	29	53	55	76	49	40	35	57	N/A

**Table 2:** Hematology Parameters. Data from complete blood count test describing the physiological hematological changes for individual pigs, days post infection (d.p.i).

Blood Parameters	Control/PBS (d.p.i)				Ad5ΔE3-Luc (d.p.i)				Ad5/3ΔE3-Luc (d.p.i)				Reference
Measurement	0	2	4	7	0	2	4	7	0	2	4	7	Range
Red Blood Cells (×10^6^/µL)	5.5	5.8	5.3	5.1	5.4	6.3	5.4	5.9	6.3	6.2	5.9	5.7	5–8
Hemoglobin (g/dL)	10.0	10.4	9.7	9.1	9.8	11.5	9.7	10.9	11.0	11.0	10.4	10.1	10–16
Hematocrit (%)	32.7	32.5	29.7	28.7	31.8	34.8	31.0	35.2	36.8	33.0	32.1	30.6	32–50
Platelets (×10^3^/µL)	357	386	300	412	131	234	400	293	260	222	361	377	325–715
White Blood Cells (×10^3^/µL)	17.5	20.9	15.0	15.5	11.2	14.0	10.7	11.0	14.0	20.6	14.5	13.2	11–22
Neutrophils (×10^3^/µL)	6.7	11.9	7.4	7.0	4.7	6.2	4.6	3.6	5.9	14.4	5.1	5.7	0.3–15.2
Lymphocytes (×10^3^/µL)	10.7	7.3	6.3	7.4	5.5	5.6	4.8	6.2	7.4	4.3	8.4	6.5	3.6–18.5
Monocytes (×10^3^/µL)	0.2	1.7	0.3	0.5	0.8	2.0	1.1	0.7	0.3	1.2	0.2	0.4	0.0–4.9
